# Purtscher-Like Retinopathy Associated with Synthetic Cannabinoid (Bonzai) Use

**DOI:** 10.4274/tjo.galenos.2018.67670

**Published:** 2019-04-30

**Authors:** Zafer Onaran, Yaprak Akbulut, Serkan Tursun, Tevfik Oğurel, Nesrin Gökçınar, Ayşegül Alpcan

**Affiliations:** 1 Kırıkkale University Faculty of Medicine, Department of Ophthalmology, Kırıkkale, Turkey; 2 Kırıkkale University Faculty of Medicine, Department of Pediatrics, Kırıkkale, Turkey

**Keywords:** Cannabinoid, substance abuse, Purtscher-like retinopathy

## Abstract

Purtscher’s retinopathy is a microvascular occlusive disease initially described as retinal edema, cotton wool-like exudation, and hemorrhages occurring after severe head trauma. A similar clinical presentation called Purtscher-like retinopathy is associated with systemic diseases instead of trauma. In the present case, ophthalmic eksamination of a patient with complaints of blurred vision related to substance (Bonzai) use revealed bilateral cotton-wool spots. Purtscher-like retinopathy was diagnosed based on fluorescein angiography and optical coherence tomography findings. This is the first case of Purtscher-like retinopathy associated with Bonzai use described in the literature.

## Introduction

Synthetic cannabinoids are drugs similar to the active ingredient of cannabis and in recent years their use has increased worldwide, including in Turkey, because they are cheap and easy to obtain. Known as Bonzai and Jamaica in Turkey, these substances affect the central nervous system by binding to cannabinoid 1 and 2 receptors and induce behavioral changes.^1^

Purtscher’s retinopathy is a microvascular obstructive disease first described as a result of severe head trauma and characterized by retinal findings of cotton-wool spots and hemorrhage.^2^ Purtscher-like retinopathy refers to a similar nontraumatic clinical presentation seen in a wide spectrum of illness including pancreatic disease, hematologic disorders, renal pathologies, and pregnancy-related conditions. Here, we present a case of Purtscher-like retinopathy and severe visual impairment together with serious systemic disorders in an adolescent with a history of synthetic cannabinoid use.

## Case Report

**Figure 1:**
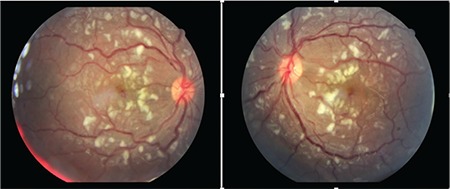
Bilateral cotton-wool spots concentrated in the posterior pole

**Figure 2:**
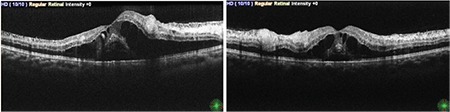
Optical coherence tomography revealed subretinal fluid, cystic macular edema, and hyperreflectivity in the ganglion cell layer corresponding to areas of soft exudate

**Figure 3:**
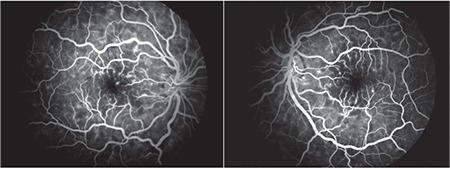
Fundus fluorescein angiography shows hyperfluorescent spots and hypofluorescent areas in regions corresponding to soft exudates

A 16-year-old patient with no known diseases presented to the pediatric outpatient clinic with muscle weakness, fatigue, nausea, vomiting, and clouding of consciousness. The patient had signs of meningeal irritation and laboratory test results showed marked elevation in aspartate aminotransferase: 2650 U/L, alanine transaminase: 1110 U/L, creatine phosphokinase: 6765 U/L, amylase: 167 U/L, and lipase: 909 U/L. Based on these results, the patient was admitted for further testing and treatment and was referred to our department due to complaints of vision loss for two days. On examination, visual acuity was 0.05 in the right eye and 0.4 in the left eye. Intraocular pressure, pupillary light reflexes, eye movements, and anterior segment examination were normal. Fundus examination revealed extensive retinal lesions consistent with the appearance of cotton-wool spots in both eyes (Figure 1). Optical coherence tomography (OCT) revealed subretinal fluid and macular edema in both eyes, and fundus fluorescein angiography showed hypofluorescent areas due to blockage and leakage from the retinal vessels in the late phase (Figures 2 and 3). Provisional diagnoses included Guillain-Barre, toxic hepatitis, hepatic encephalopathy, influenza, rhabdomyolysis, dermatomyositis, and muscular dystrophy. Detection of tetrahydrocannabinol in urinalysis and the patient’s reported use of Bonzai when questioned suggested that it was a drug-related condition. Significant abnormalities were detected in coagulation tests: complement factor C3: 41 mg/dL (↓), platelet count: 33,000 (↓), activated partial thromboplastin time: 37.5 s (↑), d-dimer: 14.9 µg/mL (↑), fibrinogen: 160 mg/dL (↑). Evaluation of the patient’s ocular findings together with the systemic presentation led to a diagnosis of Purtscher-like retinopathy. In follow-up examination 5 days later, the patient’s visual acuity had decreased further (0.05 in the right eye, 0.2 in the left eye) and macular edema showed progression in OCT. Three days after being diagnosed with retinopathy, the patient was started on megadose steroid therapy (1 g/day intravenous for 5 days) due to systemic problems, but was admitted to intensive care due to multiple organ failure and died one week later.

## Discussion

After Purtscher’s retinopathy was first described in a patient with head trauma in 1910 by Othmar Purtscher, similar clinical presentations observed in nontraumatic conditions became known as Purtscher-like retinopathy. Purtscher-like retinopathy is characterized by severe angiopathy starting within a few hours or days of the onset of systemic disease, with cotton-wool spots in the fundus, intraretinal hemorrhage, and Purtscher flecken in the acute phase. Purtscher flecken are polygonal areas of whitening in the inner retina between the retinal arterioles and venules. These lesions are pathognomonic for the disease but occur in only half of patients.^3^ Retinal edema or macular atrophy can be seen on OCT. However, OCT may be normal in some patients. Retinal ischemia, early hyperfluorescence, late leakage, peripapillary staining, and precapillary occlusion are observed on fluorescein angiography.^4^ Diagnosis is based on the patient’s history, clinical findings, and laboratory tests. Most cases are asymmetrical and bilateral, although unilateral cases are also seen.^2,5^

While Purtscher-like retinopathy can occur in acute pancreatitis, systemic lupus erythematosus, hemolysis, elevated liver enzmymes, low platelet syndrome, kidney disease, and pancreatic adenocarcinoma, it has also been associated with injection of the filler polymethyl methacrylate.^6,7^

Although there are theories regarding the pathophysiology of Purtscher-like retinopathy based on some underlying systemic diseases, it remains uncertain. The main cause is believed to be arteriole occlusion and ischemia. In patients with acute pancreatitis, proteolytic enzymes released into the systemic circulation due to pancreatic damage may induce the complement cascade and cause the formation of leukocyte, platelet, and fibrin aggregates.^8^ The elevated liver, pancreas, and muscle enzymes and abnormal coagulation parameters in our patient may explain the probable arteriolar occlusion implicated in the development of retinopathy.

There is no effective treatment specific for Purtscher’s or Purtscher-like retinopathy. Monitoring and treatment of the underlying etiology may be the most rational therapeutic option.^5^ Although there are publications on the use of megadose steroid therapy, hyperbaric oxygen therapy, and oral indomethacin in treatment, they do not provide sufficient evidence for a disease that can be self-limiting.^9^ While the benefit of intravitreal bevacizumab was reported in the treatment of macular edema associated with Purtscher-like retinopathy, the complete regression of macular edema at 3 months after injection raised questions about its effectiveness.^10^

Negative prognostic factors include choroidal hypoperfusion and involvement of the outer retinal layers, optic disc edema and leakage on angiography, history of a similar attack, and severity of systemic disease; nevertheless, vision recovers to varying degrees without any specific treatment in most cases.^2^

In the literature, conditions such as epileptic seizures, rhabdomyolysis, and liver and pancreas failure have been reported due to synthetic cannabinoids such as Bonzai. This is the first report describing the association between Purtscher-like retinopathy and substance (Bonzai) abuse, and it is important to include Bonzai use in the differential diagnosis of this disease.
